# Bitemporal Obesity: An Overlooked Sign of Visceral Obesity?

**DOI:** 10.7759/cureus.25575

**Published:** 2022-06-01

**Authors:** Mohammed Abrahim

**Affiliations:** 1 Emergency Department, Halton Healthcare, Milton, CAN

**Keywords:** visceral fat depot, facial fat, physical diagnosis, physical signs, obesity, bichat fat pad, buccal fat pad, temporal, superior temporal line, visceral adiposity

## Abstract

With increasing rates of morbidity and mortality associated with visceral obesity, as well as its related cardiometabolic disorders, physical findings that aid in diagnosing patients at risk for such conditions are extremely useful. This brief report introduces the novel facial physical sign of bitemporal obesity, which the author observed in a patient and suggests to be associated with visceral obesity.

## Introduction

Facial features have been utilized in medical practice as indicative of various distinct diagnoses such as hepatic and uremic facies. To date, there are no facial physical signs suggestive of visceral obesity. Furthermore, visceral obesity, irrespective of total body weight, is an established risk factor for cardiovascular disease, type 2 diabetes, and certain types of cancer [1]. Additionally, the risk of cardiometabolic morbidity and mortality increases with the size of visceral adipose tissue [1]. Therefore, identifying physical signs, suggestive of visceral obesity, is of great benefit. It is important to indicate that visceral fat depots are not exclusive to the abdominal cavity, they also extend into the deep planes of the face primarily in the buccal fat pad [2].

Histological examination of the buccal fat pad of the face has revealed a similar structure to abdominal visceral adipose tissue [2]. Emerging research demonstrates an association between the size of the buccal fat pad and abdominal visceral adipose tissue [3-5]. Anatomically, the buccal fat pad has four extensions, the main buccal body, pterygoid, pterygopalatine, and the temporal extension [6]. The temporal extension is termed the temporal fat pad (TFP) and is located in the temporal fossa. The TFP branches cranially from the main body of the buccal fat pad beneath the zygomatic arch into the temporal fossa [6]. Bilateral bulging of the temporal fossae could represent an enlargement of the TFP and, consequently, visceral obesity. This brief report introduces the novel facial physical sign of bitemporal obesity which the author observed in a patient with android-type obesity and suggests a potential association with visceral obesity.

## Case presentation

**Figure 1:**
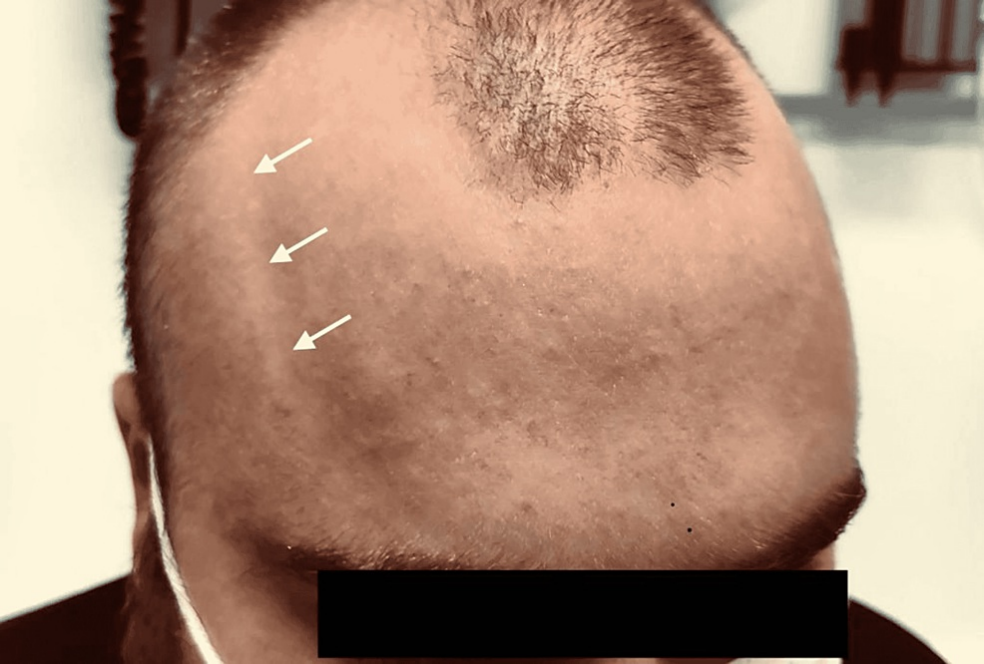
The observed temporal prominence.

A 20-year-old male presented with a minor motor vehicle collision. He was incidentally observed to have bilateral temporal prominence (Figure 1).

Such prominence is painless, symmetrical with a smooth surface, and no skin changes. Such convex temporal prominence was not related to the cause of the patient visit. He appeared to exhibit an android-type phenotype. The patient weighed 120 kg (250 lbs) at a height of 170 cm (5 ft 7 in). Furthermore, his waist circumference was 122 cm (47 in). The patient reported being healthy with no past medical history and no medication prescribed. Of particular note, he had no medical history of metabolic syndrome.

**Figure 2:**
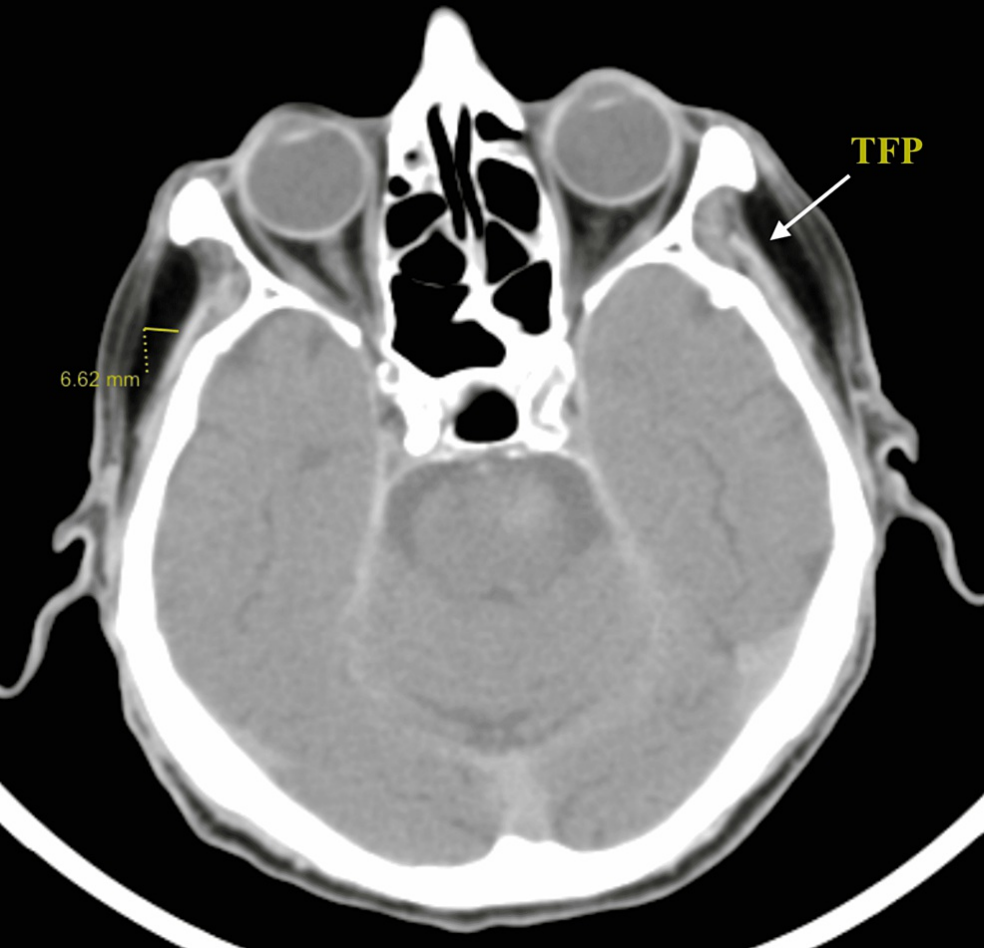
The patient’s head CT scan demonstrating a cross-sectional measurement of the temporal fat pad (TFP).

The patient’s non-contrast computerized tomography (CT) scan of the head was evaluated for the contents of the temporal fossae including TFP size. Measurements were obtained bilaterally at 6.62 mm (Figure 2).

Measurements extended from the deep surface of the superficial fascia to the superficial surface of the temporalis muscle for TFP measurement. The temporal fossae contained no masses or heterogeneous structures.

## Discussion

**Figure 3:**
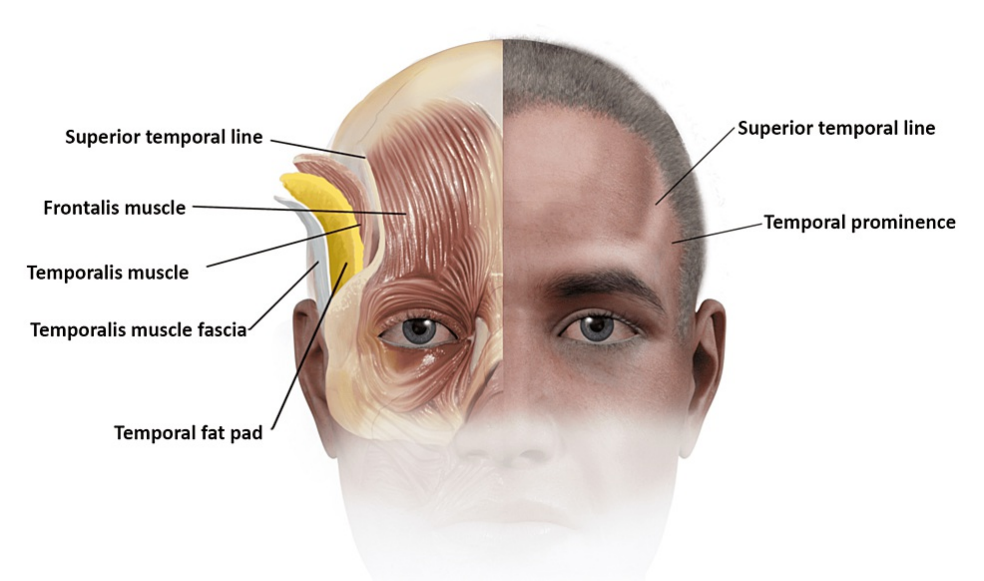
Anatomical illustration of the contents of the temporal fossa and the corresponding surface anatomy. Brittany Hand and Nicolas Fernandez created this detailed illustration.

The observed sign of bitemporal obesity corresponds to the anatomical location of the temporal fossae. The TFP occupies the middle part of the temporal fossa and is tightly encapsulated between the temporalis muscle and its deep temporal fascia; such fascia is fused to the skull creating the superior fusion line (Figure 3).

Most of the temporal fusion line is covered by hair except for its anterior portion; although, in baldness, the superior temporal line can be seen in its entirety [6]. The superior temporal lines create a characteristic two oblique sulci on the lateral aspect of the forehead.

The author observed that bitemporal obesity could represent a physical sign of visceral obesity and that this may have utility within the routine physical examination. While bitemporal obesity describes a temporal convexity, the bitemporal concavity is described as a temporal hollowing [7]. It is important to point out that temporal fat size is altered by aging, chronic corticosteroid use, hormonal replacement therapy, and following menopausal transition [8]. Further to this, unilateral temporal prominence has been previously described in patients with TFP lipoma [9,10].

To test the current observation, future volumetric studies within larger patient populations are suggested. Given that CT scan measurement of the volume of the main body of the buccal fat pad has been found to be strongly associated with the size of abdominal visceral adipose tissue, independent of the body mass index (BMI), such testing would enable further exploration of the associations between the size of the TFP and visceral fat [3-5]. Given the established association between the size of the buccal fat pad and visceral adipose tissue, it is reasonable to expect that the size of TFP, which represents the temporal extension of the buccal fat pad, also corresponds to the size of visceral adipose tissue [3-5].

Of note, TFP measurement is assessed by measuring the distance between the deep surface of the skin and the superficial surface of the temporalis muscle. However, there is a lack of standardization of the normal size of TFP within the medical literature. Since visceral obesity occurs in normal-weight individuals, observing and determining a bitemporal obesity sign could alert clinicians to investigate further the presence of visceral obesity in patients with bitemporal obesity. Subsequently, increased surveillance of bitemporal obesity could further test the hypothesis suggested herein.

## Conclusions

This case report introduces a novel physical examination sign of bitemporal obesity and suggests its potential clinical utility in guiding health professionals to detect the presence of visceral obesity. Given the associations between obesity and perturbations of the metabolism, this simple sign might also assist in identifying patients at increased risk of subsequent metabolic syndrome. Bitemporal obesity describes bilateral symmetrical prominence of the temporal fossae with a clear deep demarcation of the superior temporal line. Although a single case report cannot provide a clear demonstration of such an association, therefore further research is needed to confirm the association suggested herein. Increased awareness amongst clinicians regarding this temporal finding could aid in increasing surveillance of visceral obesity and its associated comorbidities.
